# The Potential of Stereotactic-EEG for Brain-Computer Interfaces: Current Progress and Future Directions

**DOI:** 10.3389/fnins.2020.00123

**Published:** 2020-02-27

**Authors:** Christian Herff, Dean J. Krusienski, Pieter Kubben

**Affiliations:** ^1^Department of Neurosurgery, School of Mental Health and Neurosciences, Maastricht University, Maastricht, Netherlands; ^2^ASPEN Lab, Biomedical Engineering Department, Virginia Commonwealth University, Richmond, VA, United States; ^3^Department of Neurosurgery, Maastricht University Medical Center, Maastricht, Netherlands

**Keywords:** electrocorticography, ECoG, brain-computer interface, BCI, stereotactic EEG, depth electrodes, intracranial, iEEG

## Abstract

Stereotactic electroencephalogaphy (sEEG) utilizes localized, penetrating depth electrodes to measure electrophysiological brain activity. It is most commonly used in the identification of epileptogenic zones in cases of refractory epilepsy. The implanted electrodes generally provide a sparse sampling of a unique set of brain regions including deeper brain structures such as hippocampus, amygdala and insula that cannot be captured by superficial measurement modalities such as electrocorticography (ECoG). Despite the overlapping clinical application and recent progress in decoding of ECoG for Brain-Computer Interfaces (BCIs), sEEG has thus far received comparatively little attention for BCI decoding. Additionally, the success of the related deep-brain stimulation (DBS) implants bodes well for the potential for chronic sEEG applications. This article provides an overview of sEEG technology, BCI-related research, and prospective future directions of sEEG for long-term BCI applications.

## 1. Introduction

Brain-Computer Interfaces (BCIs, Wolpaw et al., 2002) have rapidly advanced in recent years, employing a wide variety of communication and control paradigms (Huggins et al., 2017). Notably, BCIs based on electrocorticography (ECoG, Schalk and Leuthardt, 2011) have demonstrated reliable decoding of a number of cortical processes. Compared to surface electroencephalography (EEG), the superior decoding results of ECoG can be attributed to its millimeter-spatial and millisecond-temporal resolution (Parvizi and Kastner, 2018). Furthermore, ECoG is unaffected by movement artifacts and allows for the measurement of higher-frequency activity, such as the high gamma-band (>70 Hz), as it is unfiltered by dura, skull and scalp tissues. The high-gamma band might correlate with ensemble spiking (Ray et al., 2008) and contain very localized information for a variety of motor (Miller et al., 2007) (including smiling Kern et al., 2019) and speech tasks (Crone et al., 2001; Leuthardt et al., 2012).

ECoG is routinely utilized for monitoring of medication-resistant epilepsy in which the electrodes are implanted for the localization of the seizure origin. The procedure involves a craniotomy to place strips or grids of electrodes directly on the cortex. The electrodes generally remain implanted for a period of one to two weeks during which the brain signals are recorded and monitored to localize the seizure origin. The ECoG electrodes are also used for functional mapping of the eloquent cortex via electrical cortical stimulation (Arya et al., 2018). In addition to epilepsy procedures, ECoG can also be collected intraoperatively during awake craniotomies for brain tumor resection surgeries.

Patients undergoing these procedures are recruited to voluntarily participate in neuroscientific research and, more recently, BCI research. These investigations have allowed for tremendous advances both in the understanding of cortical processes as well as BCI technology. However, as ECoG electrodes are typically placed over specific, localized regions of the cortex based on the clinical needs of the patients, broad coverage is generally not achieved. Furthermore, ECoG only provides access to the cortical surface and not key deeper structures such as the hippocampus, insula, Herschl's gyrus and basal ganglia.

Another method for intracranial seizure localization employs penetrating depth electrodes that are implanted through small burr holes in the skull. These electrodes are positioned using stereotactic guidance, thus the modality is referred to as stereotactic EEG (sEEG). sEEG allows for the measurement of neural activity in deeper structures of the brain. The cortical sampling of sEEG is generally much sparser than ECoG, leading to regular combined implantation of sEEG and ECoG in the same patient. However, it is believed that sEEG alone leads to fewer surgical complications than the craniotomies required for ECoG (Iida and Otsubo, 2017). As in ECoG, the usage in epilepsy monitoring opens a window to conduct neuroscientific or BCI research with these intracranial recordings without putting any additional burden on the patient. In fact, many patients welcome participation in the experiments as a diversion from the tedium of waiting in the hospital room for the occurrence of a spontaneous seizure. While sEEG is being increasingly utilized for neuroscientific research, it has received relatively little attention for BCI research. This article provides an overview of sEEG technology, BCI-related research, and prospective future directions of sEEG for long-term BCI applications.

## 2. Stereotactic EEG

**Figure 1:**
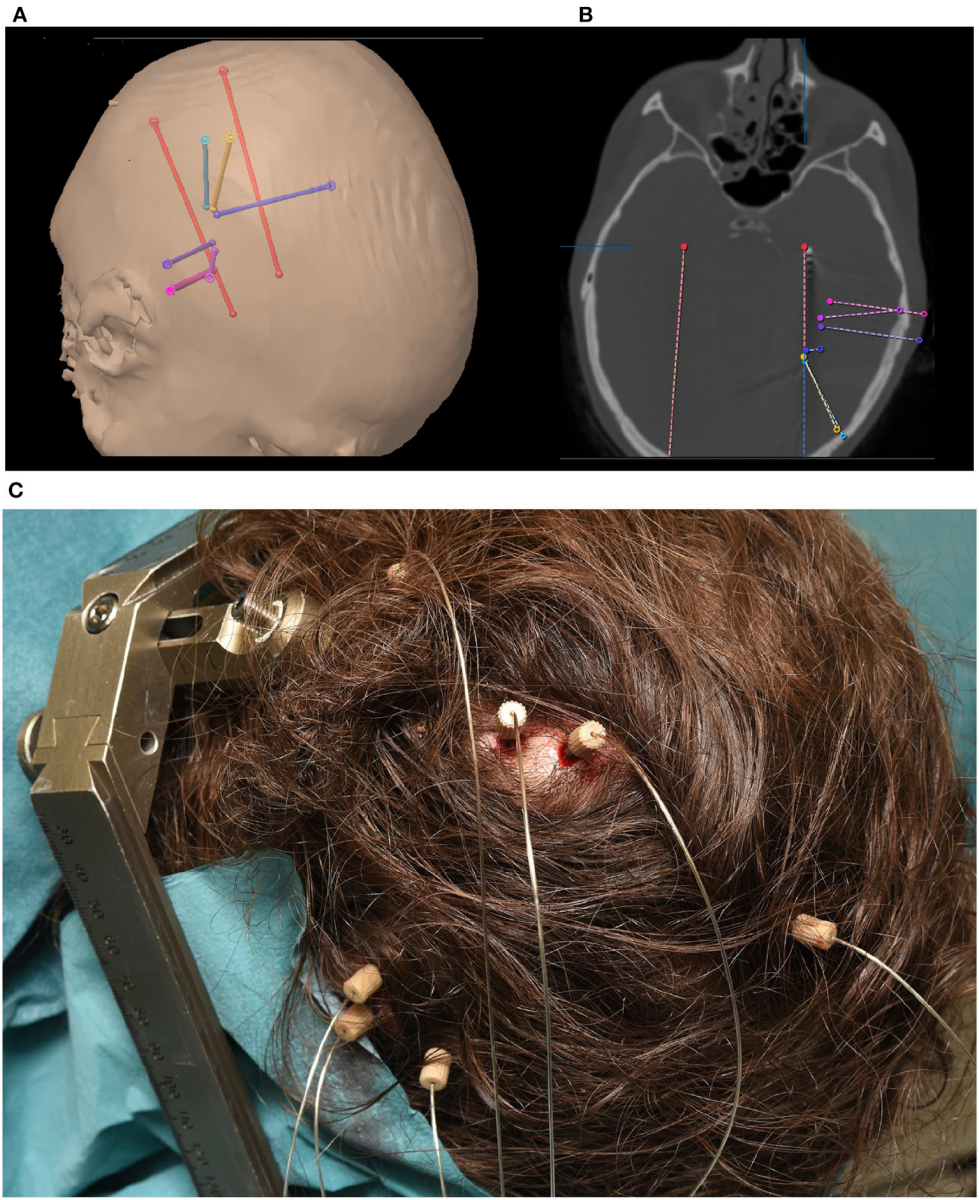
**(A)** Trajectory planning for 8 sEEG electrode shafts. **(B)** Computer Tomography showing implanted electrode shaft locations. **(C)** Implanted electrode shafts. sEEG requires only small, localized burr holes compared to the comparatively large craniotomies required for ECoG implants.

The implantation of depth electrodes guided by a stereotactic frame is called stereotactic/stereo electroencephalography (sEEG) and was first developed by Talairach and Bancaud in Paris in the late 1950s (Bancaud, 1959; Talairach and Bancaud, 1966). The procedure has become a common practice to identify epileptogenic zones in refactory elipepsy (Chassoux et al., 2018). After the patient has been identified as a candidate for invasive recordings, the epileptologist and neurosurgeon plan the trajectory of typically 5–15 cylindrical sEEG electrode shafts containing 8–18 contacts, each. Typical contacts are made from platinum/iridium, have a length of roughly 2 mm, a diameter of 1 mm and a resulting total surface area of 10 mm^2^ (suppliers include e.g., Dixi Medical, Beçanson, France and Ad-tech Medical, Oak Creek, U.S.A.). The typical inter-electrode distance is roughly 1.5–3.5 mm (van der Loo et al., 2017), which generally provides localized sampling of sparse brain regions. This can result in a total of hundreds of distinct recording sites across the brain, allowing for simultaneous recording within and across various brain structures. Less sEEG electrodes are usually implanted when sEEG is used in combination with ECoG. Figure 1 shows an example of the implantation of 8 sEEG electrode shafts.

sEEG electrodes are generally preferred over ECoG grids when the lateralization of the seizures is unknown or is expected to be in deeper brain structures, such as insula or hippocampus (Parvizi and Kastner, 2018). This preference results in regular targeting of limbic structures including the medial temporal, orbitofrontal, cingulate, and insular regions. As the electrode positioning along the trajectory spans from the skull to these deeper areas, cortical regions can also be captured. This sampling of very different areas along one electrode shaft results in special requirements for electrode referencing (Li et al., 2018). Figure 1 shows an example of a typical sEEG implantation. Red electrodes are planned (Figure 1A) to target the hippocampus and a heterotopia in the right hemisphere. Other trajectories are mainly targeting a heterotopia. Electrodes positioned along the trajectory of the planned surgical target (Figure 1B) can also capture other brain regions which can be effective for BCI applications. For example, the blue electrode trajectory is proximal to the primary motor cortex. Such coverage highlights one of the major differences between sEEG and ECoG. While ECoG provides higher density coverage over a limited cortical region (typically unilateral), sEEG provides sparser coverage spanning more, bilateral brain regions including deeper structures. As with ECoG, because the targeted areas for the electrode implants are solely determined based on clinical needs, BCI investigations in sEEG must be designed to accommodate the patient-specific montages.

Because the clinical intent is to capture epileptic activity, sampling rates between 1 and 3 kHz are commonly used, giving a temporal resolution in the sub-millisecond range. In addition to the standard frequency ranges investigated in surface EEG, namely delta (1–3 Hz), theta (4–7 Hz), alpha (8–12 Hz), beta (13–20 Hz), and gamma (21–50 Hz), sEEG allows the measurement of the high gamma band (> 70 Hz), which is highly attenuated by skull and scalp in surface EEG recordings. The high-gamma band activity has been shown to be highly correlated with task-related signals (Miller et al., 2007) and ensemble spiking of cells in the close proximity of the electrode contact (Ray et al., 2008). The high-gamma band is also known to be strongly correlated to the BOLD signal (Logothetis et al., 2001; Mukamel et al., 2005). In addition to the access to the high-gamma band, sEEG also provides higher signal amplitude (about ten times higher) and a resulting increase in Signal-Noise-Ratio up to 100 times higher (Ball et al., 2009) compared to scalp EEG. Additionally, sEEG provides very localized information, with superior spatial resolution compared to surface EEG (Parvizi and Kastner, 2018). Estimates place the number of cells measurable by an individual contact at ~500,000 (Miller et al., 2009). Artifacts such as electrocardiogram, movement artifacts and skin potentials are also significantly attenuated or even absent in sEEG recordings. While surface EEG recordings can degrade over time and show large inter-session variability due to impedance issues, intracranial recordings appear to be much more stable over extended periods of time (Chao et al., 2010).

These advantages of sEEG combined with the relative low risk profile (Cardinale et al., 2012; Hader et al., 2013; Mullin et al., 2016) associated with the small burr holes (diameter of 1.2 mm) as opposed to the full craniotomy necessary for ECoG, make sEEG a desirable modality for electrophysiological investigations. The leads employed in sEEG and the associated surgery are akin to those used for Deep Brain Stimulation (DBS) procedures, which is widely-used as a treatment for tremors, dystonia and Parkinson's Disease, with more recent application to obsessive-compulsive disorder (Greenberg et al., 2006), Tourette's syndrome (Martinez-Ramirez et al., 2018), and epilepsy (Pycroft et al., 2018). While DBS electrodes are primarily used for electric stimulation of the brain, the demonstrated long-term efficacy of chronic DBS electrodes suggests the possibility of chronic sEEG for BCI applications.

## 3. Decoding sEEG Signals for BCI

Significant BCI advances have been achieved with other intracranial (Schalk and Leuthardt, 2011) and intracortical (Bensmaia and Miller, 2014) recording modalities. Penetrating microarrays implanted on the cortex have achieved robust control of commercial tablets (Nuyujukian et al., 2018), robotic arms (Hochberg et al., 2006, 2012) and even allowed paralyzed patients to regain control of their own arms using functional electric stimulation (Ajiboye et al., 2017). ECoG arrays implanted over the cortex have achieved remarkable results in a wide variety of BCI tasks. See Schalk and Leuthardt (2011) for a review. While it is unlikely that the standard sparse sEEG implants will exhibit superior decoding performance to microarrays and ECoG for the aforementioned applications, sEEG recordings can be used in isolation or to uniquely complement these cortical recording modalities to access information from multiple sub-cortical regions. Specific regions of interest for BCI that cannot be accessed with other modalities are the limbic system and insula for memory, emotion, place cells, etc. and deeper brain regions such as the basal ganglia and subthalamic nucleus that might help to further define motor decoding. sEEG also has the unique potential to simultaneously target multiple brain networks, bilaterally. Initial investigations in the decoding of mental processes highlight the potential for targeting unique, bilateral combinations of cortical and deeper brain structures. In the following sections, we will highlight decoding results achieved with sEEG.

### 3.1. Motor BCI

A number of studies have demonstrated decoding of motor signals for BCI using sEEG. Vadera et al. (2013) demonstrate two-dimensional cursor control from depth electrodes implanted in hand and foot cortical areas. While imagined movements were not investigated, this study highlights one of the advantages of sEEG - the opportunity to record foot cortical areas that reside in the longitudinal fissure that cannot be attained with surface measurements.

Another study (Li et al., 2017b) investigates the control of a prosthetic hand using sEEG electrodes in the central sulcus. The investigators were able to decode three different hand gestures and a resting state with good accuracies. Another robotic upper limb prosthetic employed a hybrid BCI using ECoG and sEEG, eye tracking and computer vision in two patients (McMullen et al., 2014). Two recent studies investigated the decoding of grip strength for potential use in hand prosthesis. In Murphy et al. (2016), the investigators decoded the grip strength of imagined and executed grip movements from subsurface sEEG electrodes in the central sulcus and the insular cortex and conclude that “depth electrodes could be useful tools for investigating the functions of deeper brain structures as well as showing that central sulcus and insular cortex may contain neural signals that could be used for control of a grasp force BMI.” Fischer et al. (2017) also showed that beta and gamma activity in the STN is modulated depending on the level of imagined grip force. Their study is based on electrodes implanted for DBS in the treatment of Parkinson's disease.

### 3.2. Visual Speller BCI

Studies have successfully decoded different visual-evoked potentials from sEEG recordings. In Krusienski and Shih (2011) depth electrodes in and adjacent to the hippocampus were used to successfully operate a visual speller using the P300 response. With decoding accuracies at or near 100% using less than 15 visual stimulations, achieved results were similar to those achieved with ECoG (Krusienski and Shih, 2011). This performance can be attributed to the existence of the P300 in the hippocampus (McCarthy et al., 1989) and that several of the posterior electrodes were bordering the occipital lobe. Additionally, the same group showed that similar performance could also be achieved using electrodes that were located in the lateral ventricle (Shih and Krusienski, 2012). By employing a motion-onset VEP (Kuba et al., 2007) and sEEG electrodes in middle temporal regions, Li et al. (2017a) showed that up to 14 characters per minute could be typed.

### 3.3. Speech BCI

Another type of BCI that has rapidly developed are interfaces that aim to restore the ability to speak (Herff and Schultz, 2016; Schultz et al., 2017). Studies have shown that it is possible to decode ECoG activity into text (Herff et al., 2015; Moses et al., 2016, 2019) and speech output (Herff et al., 2016; Angrick et al., 2019; Anumanchipalli et al., 2019). Using depth electrodes, Chrabaszcz et al. (2019) showed that STN is also active during speech production. Two recent advances showed that decoding of speech perception from depth electrodes is also possible. In Akbari et al. (2019) perceived speech was decoded from sEEG electrodes in auditory cortex into an audible waveform. In this approach, sEEG electrodes even yielded slightly better results than ECoG recordings. In Han et al. (2019), the authors decoded the attended speaker for intelligent hearing aids. In this study, one participant was implanted with bilateral temporal depth electrodes covering left and right auditory cortex. The goal of this line of research it to be able to increase intelligibility of attended speaker for smart hearing aids.

### 3.4. Navigational BCI

The discovery of place and grid cells in the hippocampus (Maguire et al., 1998; Moser et al., 2008) has greatly advanced our understanding of human spatial navigation. As sEEG electrodes can sample from the hippocampus and epilepsy monitoring often requires electrodes in the hippocampus, an unparalleled opportunity to decode navigational parameters from sEEG activity arises. Several different aspects of navigation have been decoded from sEEG electrodes in the hippocampus. Aghajan et al. (2017) used neural networks to decode movement speed. Another study (Vass et al., 2016) showed successful decoding of teleportation distance from hippocampus, highlighting that location is well-represented in these recordings. Watrous et al. (2018) extended these findings by showing that even the navigational goal can be decoded from single united activity recorded from microelectrodes at the tip of sEEG electrodes.

### 3.5. Passive BCI

Instead of directly controlling computers, the idea of passive BCIs (Zander and Kothe, 2011) is to adapt interfaces to a user's mental state such as stress, workload, drowsiness, or emotion, which the user may or may not be consciously aware of. As sEEG targets deeper brain structures including limbic regions such as the amygdala, it is well-suited to detect and decode brain activity associated with such user states. Alasfour et al. (2019) demonstrated the classification of abstract naturalistic behavioral contexts from ECoG and sEEG recordings, which could be used to adapt interfaces to the coarse behavioral context of users in the future. Sani et al. (2018) showed that mood variations during natural behavior can be decoded from intracranial recordings (including sEEG). Their classifiers relied mostly on electrodes in limbic regions. These findings could one day help in the development of closed-loop systems to treat neuropsychiatric disorders. Yamin et al. (2017) investigate online neurofeedback in depth electrodes with a virtual reality interface. Their preliminary results show that users were able to reliably downregulate their amygdala activity.

Another aspect of cognition that could be useful for passive BCI is the encoding and retrieval of memory that could for example inform an interface which information needs to be presented again. Initial investigations highlight the feasibility of decoding aspects of memory from sEEG recordings (Song et al., 2016, 2017). Hampson et al. (2018) extended these findings and demonstrated that the typical activity pattern during successful memory encoding could also be used in stimulation to increase memory performance.

## 4. Future Directions

**Figure 2:**
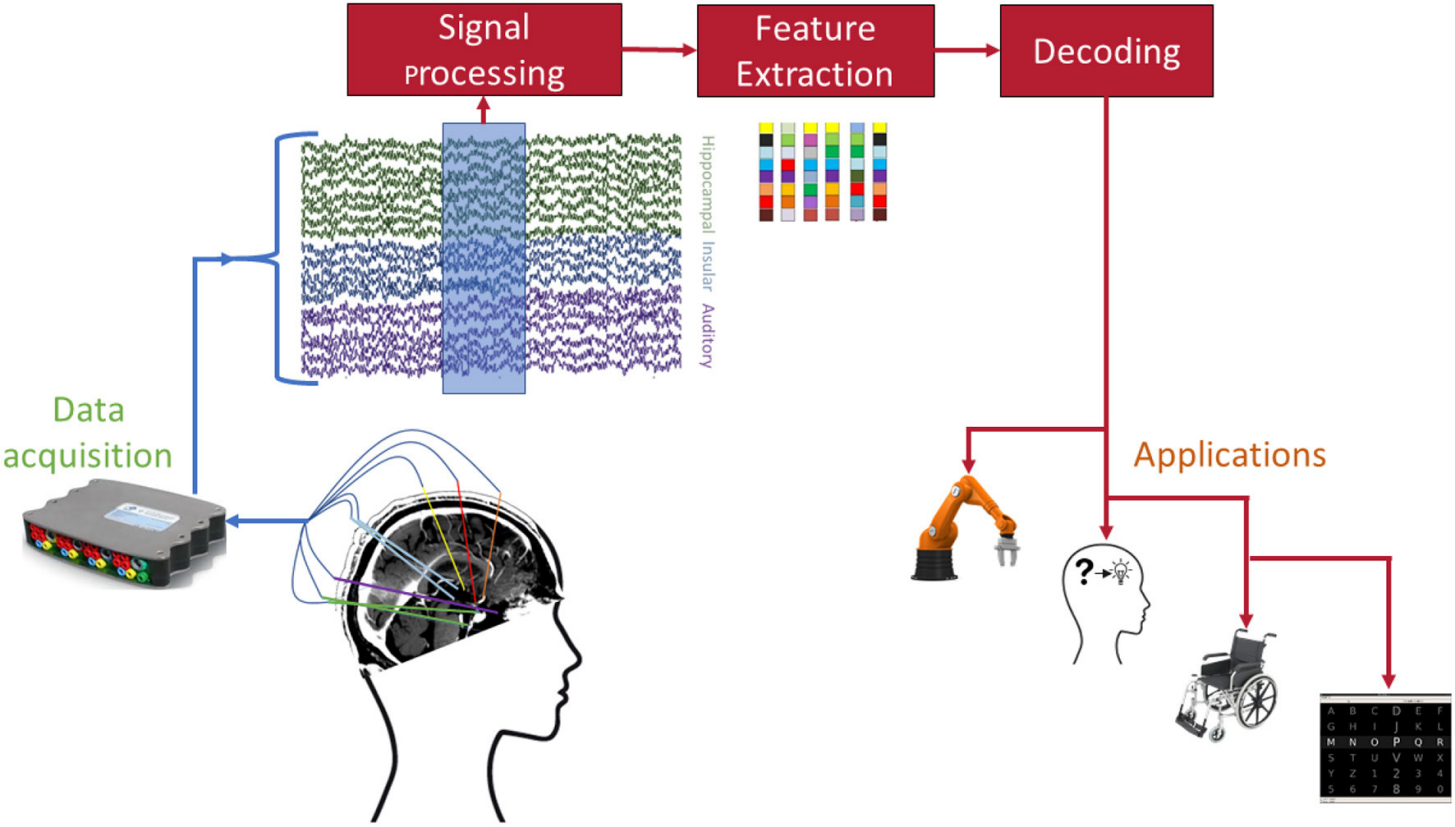
Envisioned pipeline for sEEG BCI. Each of the involved stages poses open challenges before successful dissemination to patients. Example applications include (from left to right) robotic arm control, memory prosthesis, wheelchair control, and speller interfaces.

Despite the impressive results achieved in decoding of mental processes from sEEG recordings, there are still numerous practical issues that must be addressed before sEEG BCIs can be considered for long-term, clinical applications. Figure 2 shows the standard processing pipeline of an sEEG-based BCI. At each individual stage of this pipeline there are unique challenges and opportunities for achieving a practical BCI.

For *data acquisition*, current clinical sEEG implants can be modified in a multitude of ways to improve the spatial resolution and target sampling. By maintaining the same shaft size, the contact size and density can be reduced to be able to record local field potentials along the entire length of the shaft (Pothof et al., 2016). Additionally, microwires can be placed at the tip of the shaft for recording single-units (Pothof et al., 2016). Such modifications are expected to yield significant improvements in BCI decoding performance as observed when using micro-ECoG in comparison to standard clinical ECoG (Slutzky et al., 2010; Wang et al., 2013; Kellis et al., 2016; Muller et al., 2016). Furthermore, the sEEG shafts can be designed to have custom electrode placement or directional electrodes (Tinkhauser et al., 2018) to strategically target multiple brain locations or networks using a single shaft and trajectory planning. Such sampling of multiple brain networks, including cortical and subcortical targets, would significantly increase the decoding potential for many complex functions such as language and memory. Since sEEG is well-suited for bilateral implantation, there is significant potential for investigating network coordination across hemispheres. Leveraging the clinical success of DBS based on electrical stimulation, there is also the possibility of developing bidirectional BCIs using sEEG (Wander and Rao, 2014). Additionally, the long term stability of sEEG recordings needs to be investigated. While studies show that ECoG grids provide reliable long-term measurements (Vansteensel et al., 2016; Pels et al., 2019), similar evidence for sEEG is currently lacking.

While DBS devices present fully implanted solutions, sEEG measurements still rely on externalized leads connected to bulky amplifiers. For realistic BCI applications, a fully implanted solution should be targeted placing new requirements on (wireless) amplifiers. Advances from other types of neural implants might be harnessed for these *data acquisition* challenges (Eftekhar et al., 2010; Liu et al., 2017).

The sparse sampling of sEEG across different brain regions requires specific *signal processing*, as well as *feature extraction*. For example, while high-gamma has been the focus of many intracranial BCI studies and are also found in e.g., hippocampus (Colgin and Moser, 2010), other frequency ranges such as theta might be better suited for decoding activity (Stavisky et al., 2015) from deeper structures (Buzsáki, 2002). Furthermore, sEEG provides an excellent opportunity to explore more global phenomena such as traveling waves (Nunez and Srinivasan, 2006; Muller et al., 2018), connectivity (Van Mierlo et al., 2013), and frequency-coupling (Maris et al., 2011).

In addition to the common *applications* mentioned in Figure 2, sEEG provides a unique opportunity to enhance existing or develop new applications by harnessing brain activity from limbic and memory-related brain activity. For instance, this information could conceivably be used to convey emotion or affect in a speech neuroprosthetic. As with other measurement modalities, different requirements for the *decoding* procedures will arise depending on the envisioned *application* (Borton et al., 2013; Bensmaia and Miller, 2014).

Overall, sEEG exhibits several unique advantages of other intracranial monitoring methods. In addition to the capability of sampling subcortical regions, sEEG implantation is a less traumatic procedure that exhibits a lower risk of infection. Since the hardware and procedures for sEEG and DBS implantation are effectively identical, the success and precedent established by DBS suggests that sEEG could also be chronically implanted for BCIs. Ultimately, the BCI field needs to further develop and test new sEEG electrode/shaft designs and develop paradigms that exploit sEEG's unique capability of recording from multiple cortical and subcortical targets. It is also prudent to explore sEEG in conjunction with microarrays and ECoG to evaluate whether the addition of subcortical targets and networks can further refine the decoding performance and capabilities of these already-successful approaches. It is feasible that future BCIs will require a hybrid of cortical (Microarrays and ECoG) and subcortical (sEEG) sampling on the path to achieving fully-transparent and natural operation.

## 5. Conclusion

In this review article, we briefly introduced sEEG and compared its characteristics with ECoG, another intracranial measurement modality. We reviewed initial decoding work using sEEG and highlighted further potential and future directions of BCI research using sEEG.

We believe that sEEG holds great potential for BCI as it offers the measurement of brain structures that are not reachable with ECoG and supplying a very broad sampling of neural activity. In particular, sEEG provides an unparalleled opportunity for the decoding of memory-related processes and limbic activity, which can also be incorporated to supplement or further enhance decoding of other cognitive processes.

## Author Contributions

All authors contributed to the final version of the manuscript.

### Conflict of Interest

The authors declare that the research was conducted in the absence of any commercial or financial relationships that could be construed as a potential conflict of interest.
